# (−)-*N*,*N*′-Bis[(1*S*,2*R*,5*S*)-6,6-dimethyl-bicyclo­[3.1.1]heptan-2-ylmeth­yl]pyridine-2,6-dicarboxamide monohydrate

**DOI:** 10.1107/S1600536808013652

**Published:** 2008-05-14

**Authors:** Sylvain Bernès, Francisco Javier Pérez-Flores, René Gutiérrez

**Affiliations:** a DEP Facultad de Ciencias Químicas, UANL, Guerrero y Progreso S/N, Col. Treviño, 64570 Monterrey, NL, Mexico; b Laboratorio de Síntesis de Complejos, Facultad de Ciencias Químicas, Universidad Autónoma de Puebla, A.P. 1067, 72001 Puebla, Pue., Mexico

## Abstract

The title compound, C_27_H_39_N_3_O_2_·H_2_O, is a chiral pyridine-2,6-dicarboxamide derivative including *cis*-myrtanyl groups as amine substituents. The pyridine-2,6-dicarboxamide core approximates *C*_2_ point symmetry and a solvent water mol­ecule lies on the pseudo-twofold axis. The water mol­ecule serves both as acceptor and donor for efficient hydrogen bonds involving N—H and C=O functional groups as donor and acceptor groups, respectively. As a result, each water mol­ecule in the crystal structure is tetra­hedrally bonded to three symmetry-related mol­ecules, forming a three-dimensional supra­molecular network. Such an arrangement is a common feature found in the majority of X-ray-characterized sym­metrically substituted pyridine-2,6-dicarboxamide derivatives.

## Related literature

For background to the solvent–free synthesis used for the preparation of the title compound, see: Tanaka & Toda (2000 ▶); Vázquez *et al.* (2004 ▶); Tovar *et al.* (2007 ▶); Pérez-Flores & Gutiérrez (2008 ▶). For hydrates of pyridine-2,6-dicarboxamide derivatives, see: Yu *et al.* (1999 ▶); Qi *et al.* (2002 ▶); Jain *et al.* (2004 ▶); Odriozola *et al.* (2004 ▶).
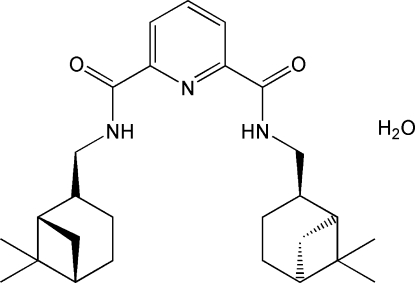


## Experimental

### 

#### Crystal data

C_27_H_39_N_3_O_2_·H_2_O
*M* *_r_* = 455.63
Monoclinic, 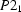

*a* = 6.8476 (11) Å
*b* = 12.1101 (14) Å
*c* = 16.012 (2) Å
β = 91.173 (15)°
*V* = 1327.5 (3) Å^3^
*Z* = 2
Mo *K*α radiation
μ = 0.07 mm^−1^
*T* = 298 (1) K
0.6 × 0.6 × 0.2 mm

#### Data collection

Bruker P4 diffractometer
Absorption correction: none
6391 measured reflections
3180 independent reflections
2580 reflections with *I* > 2σ(*I*)
*R*_int_ = 0.035
3 standard reflections every 97 reflections intensity decay: 2%

#### Refinement

*R*[*F* ^2^ > 2σ(*F* ^2^)] = 0.040
*wR*(*F* ^2^) = 0.107
*S* = 1.04
3180 reflections
319 parameters
1 restraint
H atoms treated by a mixture of independent and constrained refinement
Δρ_max_ = 0.14 e Å^−3^
Δρ_min_ = −0.13 e Å^−3^

### 

Data collection: *XSCANS* (Siemens, 1996 ▶); cell refinement: *XSCANS*; data reduction: *XSCANS*; program(s) used to solve structure: *SHELXS97* (Sheldrick, 2008 ▶); program(s) used to refine structure: *SHELXL97* (Sheldrick, 2008 ▶); molecular graphics: *ORTEP-3* (Farrugia, 1997 ▶) and *POV-RAY* (Cason, 2004 ▶); software used to prepare material for publication: *SHELXL97*.

## Supplementary Material

Crystal structure: contains datablocks I, global. DOI: 10.1107/S1600536808013652/rk2088sup1.cif

Structure factors: contains datablocks I. DOI: 10.1107/S1600536808013652/rk2088Isup2.hkl

Additional supplementary materials:  crystallographic information; 3D view; checkCIF report

## Figures and Tables

**Table 1:** Hydrogen-bond geometry (Å, °)

*D*—H⋯*A*	*D*—H	H⋯*A*	*D*⋯*A*	*D*—H⋯*A*
N2—H2⋯O3	0.82 (2)	2.16 (2)	2.941 (3)	160 (2)
N3—H3⋯O3	0.86 (3)	2.19 (3)	3.017 (3)	159 (2)
O3—H31⋯O2^i^	0.87 (5)	1.90 (5)	2.756 (3)	167 (4)
O3—H32⋯O1^ii^	0.90 (4)	1.86 (5)	2.754 (3)	171 (3)

Symmetry codes: (i) 
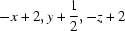
; (ii) 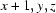.

**Fig. 1.**
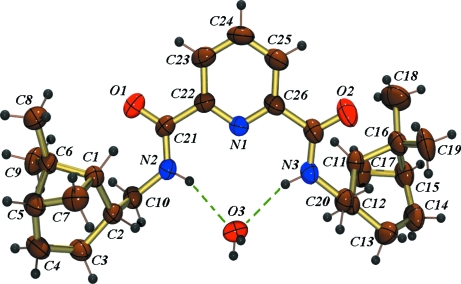
The asymmetric unit for the title compound, with the numbering scheme. Displacement ellipsoids for non–H atoms are drawn at the 40% probability level. H atoms are presented as small spheres of arbitrary radius. Dashed bonds are hydrogen bonds involving N—H groups as donor groups.

**Fig. 2.**
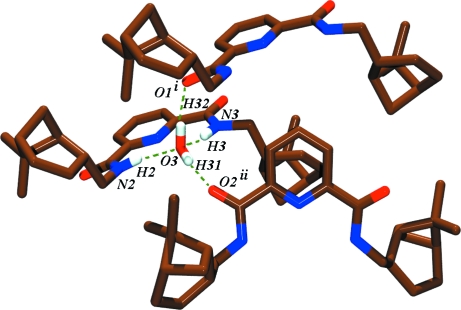
A part of the packing structure for the title compound, showing the four H bonds formed by a water molecule, in a tetrahedral geometry (dashed bonds). Symmetry codes: (i) 1+x, y, z; (ii) 2-x, 1/2+y, 2-z. H atoms not involved in hydrogen bonds have been omitted for clarity.

## supplementary crystallographic information

### Comment

Nowadays, reactions conducted in the absence of solvents under mild reaction conditions are becoming an important method in laboratories worldwide as an environment–friendly technique for the efficient syntheses of organic molecules. The main advantages of solvent-free organic synthesis are shorter reaction times, minimum waste and generally higher yields, operational simplicity as well as reduction of thermal degradative byproducts along with cleaner work–up (Tanaka & Toda, 2000). As part of an ongoing program aiming to develop simpler and eco–friendly methods for organic transformations under solvent–free conditions (Tovar *et al.*, 2007; Vázquez *et al.*, 2004), we engaged the preparation of chiral pincer ligands (Pérez-Flores & Gutiérrez, 2008). The title compound resulted from this research, by introducing chiral *cis*–myrtanyl groups as amine substituents.

The X–ray characterized monohydrate has the expected molecular geometry (Fig. 1). The pyridine–2,6–dicarboxamide core approximates a *C*_2_ point symmetry, with the pseudo 2–fold axis passing through N1 and C24. The guest water molecule O3 is placed on the pseudo 2–fold axis and is involved in two N—H···O hydrogen bonds within the asymmetric unit (Fig. 1 and Table 1, lines 1 and 2). The same water molecule is a donor group for two C═O···H intermolecular hydrogen bonds of relatively strong strength (Table 1, lines 3 and 4). As a consequence, a three–dimensional supramolecular structure is formed in the crystal structure, with water molecules being bonded in a tetrahedral arrangement (Fig. 2) to three symmetry–related molecules. Such a feature seems to be common for symmetrically substituted pyridine–2,6–dicarboxamide derivatives. These compounds are generally crystallized as hydrates, and, at least for X–ray characterized compounds, water molecules form hydrogen bonds similar to those observed in the title molecule (*e.g*. Yu *et al.*, 1999; Qi *et al.*, 2002; Jain *et al.*, 2004; Odriozola *et al.*, 2004).

### Experimental

Under solvent–free conditions, (-)-*cis*–myrtanylamine (0.38 g, 2.5 mmol) and 2,6–pyridinedicarbonyl dichloride (0.30 g, 1.5 mmol) were mixed at room temperature, giving a white solid. The crude was recrystallized from EtOH affording the corresponding dicarboxamide (98% yield).

### Refinement

C–bonded H atoms were placed in idealized positions and refined with a riding model approximation. Constrained C—H distances: 0.93 (aromatic CH), 0.96 (methyl CH_3_), 0.97 (methylene CH_2_) or 0.98 Å (methine CH). Isotropic displacement parameters: *U*_iso_= 1.5*U*_eq_(carrier C atom) for methyl groups and *U*_iso_ = 1.2*U*_eq_(carrier C atom) otherwise. Methyl groups were considered as rigid rotating groups. Other H atoms (amine groups and water molecule) were found in a difference map and refined freely. Measured Friedel pairs (287) were merged.

### Crystal data

**Table:**

C_27_H_39_N_3_O_2_·H_2_O	*D*_x_ = 1.140 Mg m^−^^3^
*M**_r_* = 455.63	Melting point = 398–401 K
Monoclinic, *P*2_1_	Mo *K*α radiation λ = 0.71073 Å
Hall symbol: P 2yb	Cell parameters from 60 reflections
*a* = 6.8476 (11) Å	θ = 4.6–12.5º
*b* = 12.1101 (14) Å	µ = 0.07 mm^−^^1^
*c* = 16.012 (2) Å	*T* = 298 (1) K
β = 91.173 (15)º	Cell measurement pressure: 101(2) kPa
*V* = 1327.5 (3) Å^3^	Plate, colourless
*Z* = 2	0.6 × 0.6 × 0.2 mm
*F*_000_ = 496	

### Data collection

**Table:**

Bruker P4 diffractometer	*R*_int_ = 0.035
Radiation source: fine-focus sealed tube	θ_max_ = 27.5º
Monochromator: Graphite	θ_min_ = 2.1º
*T* = 298(1) K	*h* = −8→6
*P* = 101(2) kPa	*k* = −1→15
ω scans	*l* = −20→20
Absorption correction: none	3 standard reflections
6391 measured reflections	every 97 reflections
3180 independent reflections	intensity decay: 2%
2580 reflections with *I* > 2σ(*I*)	

### Refinement

**Table:**

Refinement on *F*^2^	Secondary atom site location: difference Fourier map
Least-squares matrix: Full	Hydrogen site location: inferred from neighbouring sites
*R*[*F*^2^ > 2σ(*F*^2^)] = 0.040	H atoms treated by a mixture of independent and constrained refinement
*wR*(*F*^2^) = 0.107	*w* = 1/[σ^2^(*F*_o_^2^) + (0.0551*P*)^2^ + 0.0694*P*] where *P* = (*F*_o_^2^ + 2*F*_c_^2^)/3
*S* = 1.04	(Δ/σ)_max_ < 0.001
3180 reflections	Δρ_max_ = 0.14 e Å^−^^3^
319 parameters	Δρ_min_ = −0.13 e Å^−^^3^
1 restraint	Extinction correction: SHELXL97 (Sheldrick, 2008), Fc^*^=kFc[1+0.001xFc^2^λ^3^/sin(2θ)]^-1/4^
Primary atom site location: structure-invariant direct methods	Extinction coefficient: 0.037 (7)

### Fractional atomic coordinates and isotropic or equivalent isotropic displacement parameters (Å^2^)

**Table:**

	*x*	*y*	*z*	*U*_iso_*/*U*_eq_	
N1	0.6298 (3)	0.24506 (16)	0.95082 (10)	0.0520 (4)	
N2	0.5268 (3)	0.37490 (17)	0.81930 (11)	0.0558 (5)	
H2	0.636 (4)	0.374 (2)	0.8414 (14)	0.049 (6)*	
N3	0.9690 (3)	0.29137 (18)	1.03447 (12)	0.0576 (5)	
H3	0.926 (3)	0.330 (2)	0.9926 (17)	0.058 (7)*	
O1	0.2258 (2)	0.29762 (18)	0.81492 (11)	0.0731 (5)	
O2	0.9360 (4)	0.13912 (17)	1.11410 (13)	0.0893 (6)	
C1	0.4749 (3)	0.30035 (19)	0.63787 (13)	0.0529 (5)	
H1A	0.4642	0.2424	0.6803	0.063*	
C2	0.5749 (3)	0.4058 (2)	0.66831 (13)	0.0556 (5)	
H2A	0.7122	0.3869	0.6793	0.067*	
C3	0.5733 (5)	0.4954 (2)	0.59935 (16)	0.0724 (7)	
H3A	0.7040	0.5257	0.5955	0.087*	
H3B	0.4877	0.5549	0.6162	0.087*	
C4	0.5059 (6)	0.4548 (3)	0.51078 (18)	0.0866 (9)	
H4A	0.4046	0.5037	0.4893	0.104*	
H4B	0.6154	0.4589	0.4734	0.104*	
C5	0.4292 (4)	0.3386 (2)	0.51132 (15)	0.0737 (7)	
H5A	0.3843	0.3108	0.4567	0.088*	
C6	0.2858 (3)	0.3192 (2)	0.58333 (14)	0.0598 (5)	
C7	0.5754 (4)	0.2641 (2)	0.55738 (16)	0.0737 (7)	
H7A	0.7103	0.2878	0.5536	0.088*	
H7B	0.5613	0.1862	0.5445	0.088*	
C8	0.1734 (5)	0.2103 (3)	0.5733 (2)	0.0834 (8)	
H8A	0.0857	0.2152	0.5259	0.125*	
H8B	0.1003	0.1964	0.6227	0.125*	
H8C	0.2641	0.1510	0.5650	0.125*	
C9	0.1402 (4)	0.4092 (3)	0.6038 (2)	0.0831 (8)	
H9A	0.0386	0.4109	0.5617	0.125*	
H9B	0.2057	0.4793	0.6057	0.125*	
H9C	0.0843	0.3943	0.6572	0.125*	
C10	0.4962 (4)	0.45157 (19)	0.74996 (14)	0.0597 (5)	
H10A	0.5610	0.5209	0.7630	0.072*	
H10B	0.3576	0.4663	0.7430	0.072*	
C11	0.9333 (3)	0.3930 (2)	1.20404 (14)	0.0620 (6)	
H11A	0.8147	0.3610	1.1788	0.074*	
C12	1.0879 (3)	0.4242 (2)	1.14197 (14)	0.0604 (6)	
H12A	1.0340	0.4855	1.1088	0.072*	
C13	1.2723 (5)	0.4692 (3)	1.18754 (18)	0.0875 (9)	
H13A	1.3080	0.5385	1.1616	0.105*	
H13B	1.3784	0.4177	1.1788	0.105*	
C14	1.2550 (5)	0.4890 (3)	1.28285 (19)	0.0920 (10)	
H14A	1.3606	0.4507	1.3119	0.110*	
H14B	1.2692	0.5673	1.2942	0.110*	
C15	1.0623 (5)	0.4496 (3)	1.31641 (16)	0.0800 (8)	
H15A	1.0455	0.4611	1.3764	0.096*	
C16	1.0091 (4)	0.3322 (3)	1.28420 (15)	0.0737 (7)	
C17	0.8967 (5)	0.4939 (3)	1.26053 (18)	0.0884 (9)	
H17A	0.9245	0.5645	1.2346	0.106*	
H17B	0.7699	0.4942	1.2865	0.106*	
C18	0.8353 (7)	0.2817 (5)	1.3307 (2)	0.1257 (16)	
H18A	0.8757	0.2633	1.3868	0.189*	
H18B	0.7917	0.2162	1.3022	0.189*	
H18C	0.7305	0.3343	1.3322	0.189*	
C19	1.1675 (6)	0.2462 (3)	1.2809 (2)	0.0988 (11)	
H19A	1.2788	0.2762	1.2533	0.148*	
H19B	1.1201	0.1829	1.2505	0.148*	
H19C	1.2047	0.2245	1.3366	0.148*	
C20	1.1400 (3)	0.3334 (2)	1.08030 (14)	0.0637 (6)	
H20A	1.2328	0.3622	1.0409	0.076*	
H20B	1.2024	0.2731	1.1104	0.076*	
C21	0.3935 (3)	0.3035 (2)	0.84428 (13)	0.0548 (5)	
C22	0.4577 (3)	0.22560 (19)	0.91276 (13)	0.0540 (5)	
C23	0.3379 (4)	0.1377 (2)	0.93392 (16)	0.0700 (7)	
H23A	0.2180	0.1270	0.9068	0.084*	
C24	0.4017 (5)	0.0662 (3)	0.99674 (19)	0.0831 (8)	
H24A	0.3253	0.0063	1.0120	0.100*	
C25	0.5781 (5)	0.0851 (2)	1.03575 (16)	0.0756 (7)	
H25A	0.6238	0.0381	1.0777	0.091*	
C26	0.6875 (4)	0.17521 (19)	1.01182 (13)	0.0580 (5)	
C27	0.8771 (4)	0.2008 (2)	1.05701 (14)	0.0625 (6)	
O3	0.9282 (3)	0.42517 (19)	0.87612 (13)	0.0712 (5)	
H31	0.970 (6)	0.493 (4)	0.871 (2)	0.103 (12)*	
H32	1.020 (6)	0.384 (4)	0.851 (2)	0.111 (13)*	

### Atomic displacement parameters (Å^2^)

**Table:**

	*U*^11^	*U*^22^	*U*^33^	*U*^12^	*U*^13^	*U*^23^
N1	0.0609 (10)	0.0502 (9)	0.0449 (8)	−0.0055 (8)	0.0042 (7)	−0.0049 (8)
N2	0.0580 (10)	0.0596 (11)	0.0496 (9)	−0.0052 (9)	−0.0080 (8)	−0.0031 (8)
N3	0.0677 (11)	0.0598 (11)	0.0448 (9)	0.0007 (10)	−0.0059 (8)	−0.0011 (9)
O1	0.0527 (8)	0.0858 (12)	0.0805 (11)	−0.0020 (9)	−0.0054 (8)	−0.0119 (10)
O2	0.1293 (17)	0.0611 (11)	0.0760 (12)	0.0033 (12)	−0.0296 (11)	0.0112 (10)
C1	0.0608 (11)	0.0457 (10)	0.0521 (10)	0.0023 (10)	−0.0015 (9)	−0.0007 (9)
C2	0.0563 (11)	0.0551 (12)	0.0553 (11)	−0.0027 (10)	−0.0024 (9)	−0.0011 (10)
C3	0.0943 (18)	0.0577 (14)	0.0651 (14)	−0.0191 (14)	−0.0012 (13)	0.0020 (12)
C4	0.127 (2)	0.0740 (17)	0.0585 (14)	−0.0293 (18)	−0.0019 (15)	0.0070 (13)
C5	0.1024 (19)	0.0689 (16)	0.0497 (12)	−0.0215 (15)	−0.0041 (12)	−0.0041 (12)
C6	0.0670 (13)	0.0535 (12)	0.0584 (12)	−0.0066 (11)	−0.0102 (9)	0.0019 (10)
C7	0.0846 (16)	0.0659 (15)	0.0711 (15)	−0.0043 (13)	0.0138 (13)	−0.0193 (13)
C8	0.0909 (18)	0.0741 (18)	0.0846 (18)	−0.0257 (16)	−0.0117 (15)	−0.0012 (15)
C9	0.0700 (15)	0.0827 (18)	0.096 (2)	0.0101 (15)	−0.0225 (14)	−0.0004 (16)
C10	0.0726 (13)	0.0508 (12)	0.0553 (11)	−0.0009 (11)	−0.0083 (10)	−0.0044 (10)
C11	0.0634 (13)	0.0698 (15)	0.0523 (11)	0.0122 (11)	−0.0061 (9)	−0.0083 (11)
C12	0.0732 (14)	0.0547 (13)	0.0527 (11)	0.0017 (11)	−0.0121 (10)	0.0028 (10)
C13	0.0916 (19)	0.100 (2)	0.0709 (16)	−0.0322 (18)	−0.0087 (14)	−0.0056 (16)
C14	0.118 (2)	0.088 (2)	0.0689 (16)	−0.0129 (19)	−0.0287 (16)	−0.0106 (15)
C15	0.105 (2)	0.0843 (19)	0.0502 (12)	0.0075 (17)	−0.0120 (12)	−0.0118 (13)
C16	0.0944 (17)	0.0774 (18)	0.0491 (12)	−0.0003 (15)	−0.0076 (11)	0.0035 (12)
C17	0.102 (2)	0.092 (2)	0.0702 (16)	0.0267 (18)	−0.0074 (14)	−0.0248 (16)
C18	0.154 (3)	0.151 (4)	0.0734 (19)	−0.040 (3)	0.026 (2)	0.006 (2)
C19	0.145 (3)	0.0752 (19)	0.0743 (17)	0.023 (2)	−0.0426 (19)	0.0044 (16)
C20	0.0604 (12)	0.0768 (17)	0.0539 (11)	0.0021 (12)	−0.0013 (9)	−0.0068 (12)
C21	0.0500 (11)	0.0603 (12)	0.0542 (11)	−0.0010 (10)	0.0036 (8)	−0.0155 (10)
C22	0.0580 (12)	0.0564 (12)	0.0481 (10)	−0.0066 (10)	0.0098 (9)	−0.0131 (9)
C23	0.0706 (15)	0.0743 (16)	0.0654 (14)	−0.0205 (13)	0.0127 (11)	−0.0153 (13)
C24	0.101 (2)	0.0730 (17)	0.0755 (16)	−0.0347 (16)	0.0194 (15)	0.0035 (14)
C25	0.108 (2)	0.0615 (15)	0.0573 (13)	−0.0187 (15)	0.0054 (13)	0.0036 (12)
C26	0.0786 (14)	0.0501 (11)	0.0456 (10)	−0.0036 (11)	0.0059 (10)	−0.0029 (9)
C27	0.0860 (16)	0.0513 (12)	0.0501 (11)	0.0064 (12)	−0.0031 (11)	−0.0034 (10)
O3	0.0613 (10)	0.0674 (12)	0.0853 (12)	−0.0080 (10)	0.0110 (8)	−0.0047 (10)

### Geometric parameters (Å, °)

**Table:**

N1—C22	1.337 (3)	C11—C17	1.544 (4)
N1—C26	1.345 (3)	C11—C16	1.559 (3)
N2—C21	1.325 (3)	C11—H11A	0.9800
N2—C10	1.459 (3)	C12—C20	1.525 (4)
N2—H2	0.82 (2)	C12—C13	1.545 (4)
N3—C27	1.318 (3)	C12—H12A	0.9800
N3—C20	1.461 (3)	C13—C14	1.552 (4)
N3—H3	0.86 (3)	C13—H13A	0.9700
O1—C21	1.234 (3)	C13—H13B	0.9700
O2—C27	1.242 (3)	C14—C15	1.512 (5)
C1—C2	1.524 (3)	C14—H14A	0.9700
C1—C7	1.537 (3)	C14—H14B	0.9700
C1—C6	1.564 (3)	C15—C17	1.527 (4)
C1—H1A	0.9800	C15—C16	1.553 (4)
C2—C10	1.528 (3)	C15—H15A	0.9800
C2—C3	1.548 (3)	C16—C19	1.506 (5)
C2—H2A	0.9800	C16—C18	1.543 (5)
C3—C4	1.562 (4)	C17—H17A	0.9700
C3—H3A	0.9700	C17—H17B	0.9700
C3—H3B	0.9700	C18—H18A	0.9600
C4—C5	1.502 (4)	C18—H18B	0.9600
C4—H4A	0.9700	C18—H18C	0.9600
C4—H4B	0.9700	C19—H19A	0.9600
C5—C7	1.526 (4)	C19—H19B	0.9600
C5—C6	1.548 (4)	C19—H19C	0.9600
C5—H5A	0.9800	C20—H20A	0.9700
C6—C9	1.518 (4)	C20—H20B	0.9700
C6—C8	1.534 (4)	C21—C22	1.505 (3)
C7—H7A	0.9700	C22—C23	1.390 (4)
C7—H7B	0.9700	C23—C24	1.391 (4)
C8—H8A	0.9600	C23—H23A	0.9300
C8—H8B	0.9600	C24—C25	1.368 (4)
C8—H8C	0.9600	C24—H24A	0.9300
C9—H9A	0.9600	C25—C26	1.382 (4)
C9—H9B	0.9600	C25—H25A	0.9300
C9—H9C	0.9600	C26—C27	1.506 (3)
C10—H10A	0.9700	O3—H31	0.87 (5)
C10—H10B	0.9700	O3—H32	0.90 (4)
C11—C12	1.514 (4)		
			
C22—N1—C26	117.60 (19)	C20—C12—C13	111.1 (2)
C21—N2—C10	123.8 (2)	C11—C12—H12A	106.5
C21—N2—H2	119.2 (17)	C20—C12—H12A	106.5
C10—N2—H2	116.9 (17)	C13—C12—H12A	106.5
C27—N3—C20	122.3 (2)	C12—C13—C14	116.2 (3)
C27—N3—H3	120.2 (17)	C12—C13—H13A	108.2
C20—N3—H3	117.4 (17)	C14—C13—H13A	108.2
C2—C1—C7	107.55 (19)	C12—C13—H13B	108.2
C2—C1—C6	114.69 (18)	C14—C13—H13B	108.2
C7—C1—C6	87.32 (18)	H13A—C13—H13B	107.4
C2—C1—H1A	114.7	C15—C14—C13	112.7 (2)
C7—C1—H1A	114.7	C15—C14—H14A	109.1
C6—C1—H1A	114.7	C13—C14—H14A	109.1
C1—C2—C10	114.43 (19)	C15—C14—H14B	109.1
C1—C2—C3	111.28 (17)	C13—C14—H14B	109.1
C10—C2—C3	111.0 (2)	H14A—C14—H14B	107.8
C1—C2—H2A	106.5	C14—C15—C17	108.9 (3)
C10—C2—H2A	106.5	C14—C15—C16	111.8 (3)
C3—C2—H2A	106.5	C17—C15—C16	87.8 (2)
C2—C3—C4	115.1 (2)	C14—C15—H15A	115.1
C2—C3—H3A	108.5	C17—C15—H15A	115.1
C4—C3—H3A	108.5	C16—C15—H15A	115.1
C2—C3—H3B	108.5	C19—C16—C18	107.8 (3)
C4—C3—H3B	108.5	C19—C16—C15	118.8 (3)
H3A—C3—H3B	107.5	C18—C16—C15	112.3 (3)
C5—C4—C3	112.8 (2)	C19—C16—C11	121.7 (2)
C5—C4—H4A	109.0	C18—C16—C11	109.7 (3)
C3—C4—H4A	109.0	C15—C16—C11	85.0 (2)
C5—C4—H4B	109.0	C15—C17—C11	86.4 (2)
C3—C4—H4B	109.0	C15—C17—H17A	114.3
H4A—C4—H4B	107.8	C11—C17—H17A	114.3
C4—C5—C7	109.3 (2)	C15—C17—H17B	114.3
C4—C5—C6	111.9 (2)	C11—C17—H17B	114.2
C7—C5—C6	88.31 (19)	H17A—C17—H17B	111.4
C4—C5—H5A	114.8	C16—C18—H18A	109.5
C7—C5—H5A	114.8	C16—C18—H18B	109.5
C6—C5—H5A	114.8	H18A—C18—H18B	109.5
C9—C6—C8	108.1 (2)	C16—C18—H18C	109.5
C9—C6—C5	118.8 (2)	H18A—C18—H18C	109.5
C8—C6—C5	112.2 (2)	H18B—C18—H18C	109.5
C9—C6—C1	121.6 (2)	C16—C19—H19A	109.5
C8—C6—C1	109.9 (2)	C16—C19—H19B	109.5
C5—C6—C1	84.67 (18)	H19A—C19—H19B	109.5
C5—C7—C1	86.32 (19)	C16—C19—H19C	109.5
C5—C7—H7A	114.3	H19A—C19—H19C	109.5
C1—C7—H7A	114.3	H19B—C19—H19C	109.5
C5—C7—H7B	114.3	N3—C20—C12	112.38 (19)
C1—C7—H7B	114.3	N3—C20—H20A	109.1
H7A—C7—H7B	111.4	C12—C20—H20A	109.1
C6—C8—H8A	109.5	N3—C20—H20B	109.1
C6—C8—H8B	109.5	C12—C20—H20B	109.1
H8A—C8—H8B	109.5	H20A—C20—H20B	107.9
C6—C8—H8C	109.5	O1—C21—N2	124.4 (2)
H8A—C8—H8C	109.5	O1—C21—C22	119.8 (2)
H8B—C8—H8C	109.5	N2—C21—C22	115.86 (18)
C6—C9—H9A	109.5	N1—C22—C23	122.9 (2)
C6—C9—H9B	109.5	N1—C22—C21	117.59 (19)
H9A—C9—H9B	109.5	C23—C22—C21	119.5 (2)
C6—C9—H9C	109.5	C22—C23—C24	118.4 (2)
H9A—C9—H9C	109.5	C22—C23—H23A	120.8
H9B—C9—H9C	109.5	C24—C23—H23A	120.8
N2—C10—C2	111.88 (19)	C25—C24—C23	119.2 (3)
N2—C10—H10A	109.2	C25—C24—H24A	120.4
C2—C10—H10A	109.2	C23—C24—H24A	120.4
N2—C10—H10B	109.2	C24—C25—C26	118.9 (3)
C2—C10—H10B	109.2	C24—C25—H25A	120.5
H10A—C10—H10B	107.9	C26—C25—H25A	120.5
C12—C11—C17	108.1 (2)	N1—C26—C25	123.0 (2)
C12—C11—C16	115.7 (2)	N1—C26—C27	117.2 (2)
C17—C11—C16	86.95 (19)	C25—C26—C27	119.7 (2)
C12—C11—H11A	114.3	O2—C27—N3	123.5 (2)
C17—C11—H11A	114.3	O2—C27—C26	119.6 (2)
C16—C11—H11A	114.3	N3—C27—C26	116.9 (2)
C11—C12—C20	114.9 (2)	H31—O3—H32	103 (4)
C11—C12—C13	110.7 (2)		
			
C7—C1—C2—C10	−178.2 (2)	C14—C15—C16—C18	168.8 (3)
C6—C1—C2—C10	86.5 (2)	C17—C15—C16—C18	−81.7 (3)
C7—C1—C2—C3	54.9 (3)	C14—C15—C16—C11	−81.9 (2)
C6—C1—C2—C3	−40.3 (3)	C17—C15—C16—C11	27.6 (2)
C1—C2—C3—C4	−10.1 (4)	C12—C11—C16—C19	−39.6 (4)
C10—C2—C3—C4	−138.8 (3)	C17—C11—C16—C19	−148.2 (3)
C2—C3—C4—C5	7.6 (4)	C12—C11—C16—C18	−166.7 (3)
C3—C4—C5—C7	−50.0 (4)	C17—C11—C16—C18	84.7 (3)
C3—C4—C5—C6	46.1 (4)	C12—C11—C16—C15	81.3 (3)
C4—C5—C6—C9	40.2 (3)	C17—C11—C16—C15	−27.3 (2)
C7—C5—C6—C9	150.4 (2)	C14—C15—C17—C11	84.4 (3)
C4—C5—C6—C8	167.5 (3)	C16—C15—C17—C11	−27.8 (2)
C7—C5—C6—C8	−82.3 (2)	C12—C11—C17—C15	−88.3 (2)
C4—C5—C6—C1	−83.1 (2)	C16—C11—C17—C15	27.7 (2)
C7—C5—C6—C1	27.07 (17)	C27—N3—C20—C12	−97.3 (3)
C2—C1—C6—C9	−39.5 (3)	C11—C12—C20—N3	55.0 (3)
C7—C1—C6—C9	−147.6 (3)	C13—C12—C20—N3	−178.4 (2)
C2—C1—C6—C8	−167.1 (2)	C10—N2—C21—O1	−4.0 (3)
C7—C1—C6—C8	84.8 (2)	C10—N2—C21—C22	175.63 (19)
C2—C1—C6—C5	81.2 (2)	C26—N1—C22—C23	0.4 (3)
C7—C1—C6—C5	−26.87 (18)	C26—N1—C22—C21	−179.78 (18)
C4—C5—C7—C1	85.2 (3)	O1—C21—C22—N1	−170.6 (2)
C6—C5—C7—C1	−27.51 (18)	N2—C21—C22—N1	9.8 (3)
C2—C1—C7—C5	−87.9 (2)	O1—C21—C22—C23	9.3 (3)
C6—C1—C7—C5	27.22 (18)	N2—C21—C22—C23	−170.4 (2)
C21—N2—C10—C2	−95.3 (3)	N1—C22—C23—C24	−0.9 (4)
C1—C2—C10—N2	62.7 (2)	C21—C22—C23—C24	179.2 (2)
C3—C2—C10—N2	−170.3 (2)	C22—C23—C24—C25	0.5 (4)
C17—C11—C12—C20	−179.3 (2)	C23—C24—C25—C26	0.3 (4)
C16—C11—C12—C20	85.2 (3)	C22—N1—C26—C25	0.6 (3)
C17—C11—C12—C13	53.8 (3)	C22—N1—C26—C27	−177.24 (19)
C16—C11—C12—C13	−41.6 (3)	C24—C25—C26—N1	−0.9 (4)
C11—C12—C13—C14	−8.2 (4)	C24—C25—C26—C27	176.8 (3)
C20—C12—C13—C14	−137.1 (3)	C20—N3—C27—O2	−6.1 (4)
C12—C13—C14—C15	5.6 (5)	C20—N3—C27—C26	171.8 (2)
C13—C14—C15—C17	−48.7 (4)	N1—C26—C27—O2	179.6 (2)
C13—C14—C15—C16	46.6 (4)	C25—C26—C27—O2	1.8 (3)
C14—C15—C16—C19	41.7 (3)	N1—C26—C27—N3	1.6 (3)
C17—C15—C16—C19	151.2 (3)	C25—C26—C27—N3	−176.2 (2)

### Hydrogen-bond geometry (Å, °)

**Table:**

*D*—H···*A*	*D*—H	H···*A*	*D*···*A*	*D*—H···*A*
N2—H2···O3	0.82 (2)	2.16 (2)	2.941 (3)	160 (2)
N3—H3···O3	0.86 (3)	2.19 (3)	3.017 (3)	159 (2)
O3—H31···O2^i^	0.87 (5)	1.90 (5)	2.756 (3)	167 (4)
O3—H32···O1^ii^	0.90 (4)	1.86 (5)	2.754 (3)	171 (3)

Symmetry codes: (i) −*x*+2, *y*+1/2, −*z*+2; (ii) *x*+1, *y*, *z*.
